# An Evaluation of the Use of Smartphones to Communicate Between Clinicians: A Mixed-Methods Study

**DOI:** 10.2196/jmir.1655

**Published:** 2011-08-29

**Authors:** Robert Wu, Peter Rossos, Sherman Quan, Scott Reeves, Vivian Lo, Brian Wong, Mark Cheung, Dante Morra

**Affiliations:** ^1^University Health NetworkDivision of General Internal MedicineToronto, ONCanada; ^2^Centre for Innovation in Complex CareUniversity Health NetworkToronto, ONCanada; ^3^University Health NetworkDepartment of MedicineToronto, ONCanada; ^4^Centre for Faculty DevelopmentLi Ka Shing International Health Care Education CentreSt Michael's HospitalToronto, ONCanada; ^5^Keenan Research CentreLi Ka Shing Knowledge Institute of St Michael’s HospitalToronto, ONCanada; ^6^Wilson Centre for Research in EducationUniversity Health NetworkToronto, ONCanada; ^7^Department of PsychiatryUniversity of TorontoToronto, ONCanada; ^8^Sunnybrook Health Sciences CentreDepartment of MedicineToronto, ONCanada; ^9^Centre for Interprofessional EducationUniversity of TorontoToronto, ONCanada

**Keywords:** Email, cellular phone, interdisciplinary communication, hospital communication systems

## Abstract

**Background:**

Communication between clinicians is critical to providing quality patient care but is often hampered by limitations of current systems. Smartphones such as BlackBerrys may improve communication, but studies of these technologies have been limited to date.

**Objective:**

Our objectives were to describe how smartphones were adopted for clinical communication within general internal medical wards and determine their impact on team effectiveness and communication.

**Methods:**

This was a mixed-methods study that gathered data from the frequency of smartphone calls and email messages, clinicians' interviews, and ethnographic observations of clinical communication interactions. Triangulation of qualitative and quantitative data was undertaken to develop common themes that encompass comprehensive and representative insights across different methods.

**Results:**

Findings from our study indicated that over a 24-hour period, nurses sent on average 22.3 emails to the physicians mostly through the “team smartphone,” the designated primary point of contact for a specific medical team. Physicians carrying the team smartphone received on average 21.9 emails and 6.4 telephone calls while sending out 6.9 emails and initiating 8.3 telephone calls over the 24-hour period. Our analyses identified both positive and negative outcomes associated with the use of smartphones for clinical communication. There was a perceived improvement in efficiency over the use of pagers for clinical communication for physicians, nurses, and allied health professionals. In particular, residents found that the use of smartphones helped to increase their mobility and multitasking abilities. Negative outcomes included frequent interruptions and discordance between what doctors and nurses considered urgent. Nurses perceived a worsening of the interprofessional relationships due to overreliance on messaging by text with a resulting decrease in verbal communication. Unprofessional behaviors were observed in the use of smartphones by residents.

**Conclusions:**

Routine adoption of smartphones by residents appeared to improve efficiency over the use of pagers for physicians, nurses, and allied health professionals. This was balanced by negative communication issues of increased interruptions, a gap in perceived urgency, weakened interprofessional relationships, and unprofessional behavior. Further communication interventions are required that balance efficiency and interruptions while maintaining or even improving interprofessional relationships and professionalism.

## Introduction

Effective communication between clinicians to coordinate patient care is critical for providing quality health care to patients [1]. Frequent interruptions through paging is a major communication issue [2-4], and poor communication can result in inefficiencies and errors [5-7]. The burden from inefficient communication has been well documented in multiple areas of hospital care, and a systematic review has linked interruptions to medical errors [8-12]. 

The use of smartphones such as BlackBerrys may improve hospital communication as they provide multiple communication modalities [3]. For example, with urgent issues, direct calling can eliminate the need to wait for a page to be answered. In contrast, for nonurgent issues, asynchronous communication through email can be used, which could reduce disruptions. While many clinicians own cellular phones and smartphones, their use for clinical communication is variable [13,14]. Other than two studies that reported a perceived improvement in workflow efficiency from clinicians’ surveys, the study of smartphones used in hospital communication has been limited [15,16]. 

To understand the impact of the use of smartphones on the delivery of hospital care, we conducted a mixed-methods study to describe how smartphones were used, to identify advantages and disadvantages associated with their use, and to determine how their use can be improved.

## Methods

### Intervention 

Beginning in March 2008, each resident on the general internal medicine units received an individual BlackBerry smartphone to use for clinical communication typically within or among the medical teams [15]. BlackBerry devices were selected because of the secure email functionality and because these were the standard smartphones used by hospital administration. In addition, each team also had a “team BlackBerry” that was designated as the primary point of contact for nurses and allied health professionals to communicate with the teams. The team BlackBerry was typically carried during the day by the senior resident and then given to the covering junior resident during sign over. Specifically, nurses would contact residents by sending emails to the team BlackBerry with the following structures and information: (1) the patient’s name, (2) the nurse’s name, (3) the issue and purpose of contact, and (4) their preferred response (callback, email, or no response required). For urgent patient issues, nurses and other clinicians were asked to call the team BlackBerry directly. For nonurgent issues, clinicians were asked to contact the team BlackBerry through email. This recommendation was based on previous findings in which a high number of direct calls were found to be very disruptive for residents [15].

### Design

A mixed-methods approach was adopted to obtain a variety of data sources on the communication processes occurring on the wards. The following three methods were used: (1) quantitative measures assessing the frequency and use of smartphone calls and email messaging, (2) semistructured interviews with clinicians, and (3) ethnographic observations of clinical communication interactions were conducted using two techniques: (1) nonparticipatory “work shadowing” (defined below) and (2) observations at the nursing stations.

The settings were four general internal medicine wards at two large urban teaching hospitals in Canada. At each site, there were four medical teams each consisting of an attending physician, a senior resident, junior residents, and medical students. The study was conducted from January 5, 2009, until May 28, 2010.

### Data Collection

Between January 5, 2009 and May 28, 2010, quantitative and qualitative data were collected on communication patterns from in-depth interviews and from observations of communications.

#### Communication Patterns

Quantitative data on the usage of the smartphones to receive and place telephone calls and emails were gathered by accessing email accounts and phone records of consenting residents. A total of 12,936 emails and 13,717 phone calls were analyzed from 34 residents (Table 1).

#### In-depth Interviews

Semistructured interviews were conducted to explore clinicians’ perceptions of their experiences using the smartphones. To conduct qualitative comparisons and to ensure that a variety of clinicians’ perspectives were represented, we adopted a purposive sampling strategy where different groups of health care professionals with differing views on the use of smartphones for clinical communications were interviewed (Table 1). Each interview lasted between 15 and 40 minutes and was carried out at a mutually convenient location within the two hospital sites. The interviews were conducted and audio taped by an independent research associate and then professionally transcribed before analysis. The interview protocol consisted of a series of open-ended questions with appropriate follow-up probes that focused on users’ perspectives and their experiences using the smartphone technology. 

#### Observations of Communications

Ethnographic observational methods were employed to explore and understand the in-depth communication processes and behaviors around smartphone use [17]. Field notes included recording the usage on the types of communication tools used as well as communication interactions and incidents between clinicians. Two types of observational methods were used. First, a nonparticipatory “work-shadowing” approach was employed whereby a researcher followed medical residents for 2- to 5-hour periods during the day and evening shifts starting from 10 am to 11 pm. Both incoming and outgoing communications were recorded during these shifts. Second, observations were also conducted for 2-hour periods at the general internal medicine nursing stations, the hubs of interprofessional communication starting from 10 am and lasting until 10 pm (Table 1).

The study was approved by the Research Ethics Board, University Health Network, Toronto, Ontario.

**Table 1:** Breakdown on the data methods by data collection

Data Methods		Site 1	Site 2	Total
**Blackberry usage**				
	Residents, n	17 residents	17 residents	34 residents
	Emails, n	3946 emails	8990 emails	12,936 emails
	Phone calls, n	5714 calls	8003 calls	13,717 calls
**Semi structured interviews**				
	By attending physicians, n	4	0	4
	By medical residents, n	3	1	4
	By nurses, n	8	7	15
	By allied health professionals (pharmacists, social workers, occupational therapists), n	7	1	8
**Work shadowing**				
	Senior residents, (n) time observed (h:min)	(5) 24 h:44 min	(2) 9 h:33 min	(7) 34 h:17 min
	Junior residents, (n), time observed (h:min)	(5) 24 h:11 min	(2) 8 h:13 min	(7) 32 h:24 min
	Total residents, (n), time observed (h:min)	(10) 48 h:55 min	(4) 17 h:46 min	(14) 66 h: 41 min
	Day shifts (10 am to 6 pm): total hours observed (h:min)	34 h:36 min	12 h:48 min	47 h:24 min
	Evening shifts (5 pm to 11:30 pm): total hours observed (h:min)	14 h:19 min	4 h:58 min	19 h:17 min
**Ward observations at nursing stations**				
	Number of nursing wards sampled	2	2	4
	Number of observation sessions conducted	21	15	36
	Total hours observed (h:min)	42 h:28 min	29 h:51 min	72 h:19 min
	Hours observed weekdays, daytime (10 am to 6 pm) (h:min)	24 h:23 min	17 h:51 min	42 h:14 min
	Hours observed,weekdays, evening (6 pm to 10 pm ) (h:min)	6 h:3 min	8 h:0 min	14 h:3 min
	Hours observed weekend, daytime (10 am to 6 pm) (h:min)	12 h:2 min	4 h:0 min	16 h:2 min

### Analysis 

To determine communication volume using smartphones, descriptive statistics of calls and emails per day were calculated from email and call logs. Incoming and outgoing communications recorded during work-shadowing sessions were also identified and descriptive statistics of the different communication methods per hour were calculated. All emails sent by the consenting residents were analyzed to determine the frequency of emails that were received by others such as nurses, attending physicians, or allied health providers. Similarly, all emails received by consenting residents were analyzed to determine the frequency of emails received from different types of senders.

Interviews were transcribed, and inductive thematic analysis was performed using the qualitative data analysis software NVivo 8 (QSR international, Doncaster, Victoria, Australia). The transcripts were coded by three members of the research team (authors RW, SR, and VL) to derive and identify a number of common perceptions and broad themes.

For the ethnographic methods, the number and types of communication events occurring during the work-shadowing sessions were tabulated. Field notes from both the work-shadowing and ward observations were reviewed to identify common themes.

Triangulation of qualitative and quantitative data was undertaken to develop themes that encompass comprehensive and representative insights that are common across multiple methods. 

## Results

Key results are presented in two main sections. First quantitative data reporting smartphone communication volume is described. Second, qualitative data describing perceptions and ethnographic fieldwork is presented.

### Description of Communication Volume

**Figure 1:**
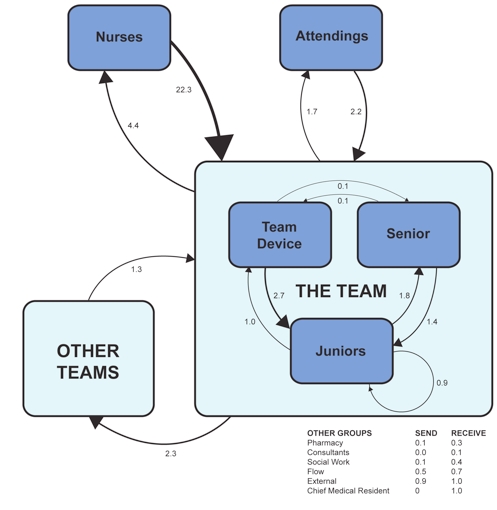
Email communication groups and frequency (average emails/day)

The usage of smartphones for telephone calls is shown in Table 2. Outgoing calls were placed to the hospital 41.2% of the time, to another BlackBerry 25.3% of the time, and to external numbers 33.4% of the time. From the 12,936 emails that were sent from or received from the smartphones, the daily frequencies of who communicates with whom were calculated (Figure 1). 

The different incoming and outgoing communications observed during work shadowing of residents are listed in Table 2. Note that these numbers are different from those calculated from device usage as work shadowing typically occurred at busier times during the day and evening.

**Table 2:** Communications to and from smartphones based on usage and work-shadowing data

		Modes of Communication						
		Telephone Calls Mean (SD)		Emails^1^ Mean (SD)		Pages^2^ Mean (SD)	Face-to-Face Conversations Mean (SD)	
Communication Processes		Received	Initiated	Received	Initiated	Initiated	Received	Initiated
**From analysis of device usage**								
	Team smartphones per 24-hour period	6.4/24h (5.3)	8.3/24h (6.4)	21.9/24h (10.1)	6.9/24h (4.8)	Not Applicable	Not Applicable	Not Applicable
	Senior smartphones per 24-hour period	5.2/24h (3.9)	5.3/24h (5.0)	3.3/24h (2.8)	2.1/24h (2.2)	Not Applicable	Not Applicable	Not Applicable
	Junior smartphones per 24-hour period	3.9/24h (3.2)	5.4/24h (5.1)	3.6/24h (3.9)	2.4/24h (3.5)	Not Applicable	Not Applicable	Not Applicable
**From analysis of work-shadowing observation**								
	Resident communications per hour	1.1/h (1.2)	1.4/h (0.8)	1.7/h (1.8)	1.0/h (1.1)	0.7/h (0.8)	1.8/h (0.8)	2.3/hr (1.4)

^1^Emails include regular emails as well as short messaging services messages.

^2^Since only residents on general medicine were given smartphones, paging was typically used to contact other services or medical students who did not have smartphones.

### Themes

The analysis of qualitative data (interviews, work shadowing, and ward observations) generated five major themes: efficiency, interruptions, interprofessional relations, gaps in perceived urgency, and professionalism (Table 3).

**Table 3:** Themes with number of participants and number of occurrences by data collection method

		Data Collection Method		
		Interviews	Work Shadowing Residents	Ward Observations
Key themes				
**Theme 1: Efficiency**		n participants (n quotes)	n participants (n incidents)	n participants (n incidents)
	Improved efficiency	8 physicians (38) 11 nurses (36) 5 allied health (23)	12 residents (39)	23 clinicians (58)
	Reduced efficiency	3 physicians (8) 9 nurses (16) 1 allied health (3)	3 residents (4)	3 clinicians (4)
**Theme 2: interruptions**				
	Increased Interruptions	7 physicians (34) 4 nurses (7) 1 allied health (3)	13 residents (46)	7 clinicians (8)
**Theme 3: interprofessional relationships**				
	Improved interprofessional collaboration	5 physicians (9) 3 nurses (3) 2 allied health (2)	0 residents (0)	0 clinicians (0)
	Reduced interprofessional collaboration	3 physicians (4) 9 nurses (27) 1 allied health (2)	3 residents (3)	8 clinicians (15)
**Theme 4: gaps in perceived urgency**				
	Differing standards for when emails or direct calls should be used	6 physicians (20) 12 nurses (22) 2 allied health (3)	2 residents (2)	15 clinicians (25)
**Theme 5: professionalism**				
	Perceived lack of etiquette for answering calls or texting on smartphone devices	2 physicians (9) 2 nurses (3) 3 allied health (10)	8 residents (16)	1 clinicians (1)

### Efficiency

#### Nurses and Allied Health Professionals

With the smartphone system, nurses no longer felt the need to wait for a telephone reply, which typically was required with paging, and this resulted in less “phone tag.” Some nurses and allied health professionals perceived a faster response and increased accessibility to physicians (Textbox 1, data extract 1). They found the use of emails helped to convey their patient’s status quickly and efficiently to doctors (Textbox 1, data extract 2). Nurses also reported that since their emails were sent to the team BlackBerry, less time was spent trying to locate a specific resident (Textbox 1, data extract 3). Some of the nurses interviewed appreciated that the smartphone system allowed direct and immediate communication by phone with physicians for urgent issues. Direct calls were observed to be from nurses or other clinicians who previously were unsuccessful at getting a response from a resident through email or paging (Textbox 1, data extract 4).

A number of the nurses interviewed perceived that the new system added a barrier, with increased difficulty reaching doctors. Instead of being able to resolve complex issues quickly with a page coupled with a brief discussion over a telephone call as done in the old system, nurses found it unproductive to have multiple emails being sent back and forth (Textbox 1, data extract 5). Instead, nurses found that telephone or face-to-face conversations allowed more detailed discussions compared with the short text of an email.

#### Residents

Residents also perceived significant efficiency with the use of smartphones. Since 42% of emails from nurses were informational items, no follow up response was required for these types of communication (Textbox 1, data extract 6). Residents also used the smartphones to reduce the inefficiencies of having to page other services to a ward telephone. By paging other physicians to their smartphone, they no longer needed to wait at a ward telephone for a callback and were able to perform other tasks while waiting for the return call (Textbox 1, data extract 7). To the residents, smartphones made it easier to coordinate activities within the teams through email or telephone calls (Textbox 1, data extract 8). The devices were also used to communicate with other physicians from other teams and services to increase communication around patients (Textbox 1, data extract 9). Residents appeared to incorporate the phone and messaging functions of the smartphone in their clinical work, using them to call a patient's family member, to communicate rapidly with other team members, and to respond to urgent situations (Textbox 1, data extract 10).

**Efficiency: extracts from interviews and field notes**

Interview, nurse 13 1. I think the message is getting across better. The communication has been opened a lot so you don’t have to always sit there on the phone and then they call back and you miss the phone call and then you have to call again. The information is directly there so they know exactly what it is if they want to respond to it immediately or when they have time.

Field notes, ward observations, Nov 27th 2. At 10:01:01, a nurse types [an email]: “Team 1: Call back requested--Message: pt continues to experience chest pain. Last 0.4mg nitro spray given at 8:30. Patient rating pain as 5 out of 10. Will order ECG. Please call unit.”

Interview, nurse 1 3. A nurse would come to the nursing station and say, “Mrs. Jones’ potassium is 2.7. Can you page the resident that’s looking after this patient?” Now it makes it easier for us because we no longer have to search on the whiteboard or through the chart to see who is the covering physician because we just send out a general email to team (number) and the person who is carrying the BlackBerry will deal with this issue. They’ll either forward it to the right person or they’ll deal with it themselves.

Field notes, ward observations, Nov 2nd 4. At 2:43, nurse 2 came to the ward clerk. She mentioned that no one was responding after 2 [emails] and so she is going to page. She first paged and then she called another person (name M) to leave a message that patient X’s ECG abnormal results are back and want doctor 1 to know. After hanging up, she realized she could call the BlackBerry. “Oh…forgot about it. There are many ways to reach the doctor.” She called doctor 1 on the BlackBerry. It turns out the doctor was at the stairway on his way up. Doctor 1 arrived at nursing station and said, “Sorry that I didn’t call you back.” Discussion for patient’s case commences between nurse 2 and doctor 1.

Interview, nurse 1 5. ...because maybe I just find sometimes some additional problems come up again and you have to be able to go through the whole system.... But I find that like if I read through the emails, they’ve emailed each other back several times. So if you’re just able to pick up the phone and call them. I personally think that I’ve always been pro talking to someone [rather] than just technology.

Interview, resident 4 6. I really liked using the BlackBerrys because it very quickly communicated what the nurse was trying to contact...

Interview, resident 2 7. Especially when you are trying to get in touch with specialists. You don’t have to stick around the telephone to wait for a phone call back. You can do your things and the specialist can call you at his convenient time.

Interview, resident 1 8. When your team needed to get together to discuss something or to run the list or whatever you’d send out five pages, which is ridiculous. You’d have to sit at the phone and wait for five people to call you back. This way you send one message to five people that says “Meeting on 13 in five minutes.” And everyone just comes. ”

Interview, resident 1 9. Oftentimes I’ll be consulting with another physician on a patient and I’ll say, “This is my BlackBerry. Call me back after you’ve seen the patient or call me back when you have a plan.”…So that’s extremely valuable, which we never had with pages, and no one would ever page you for that because it was too much of a pain.

Field notes, work shadowing physician 4 10. At 10:23, team BlackBerry goes off. Senior picks up and talks. She hangs up at 10:24 and goes to the elevator. She looks at the team BlackBerry and starts typing. She then calls using her senior’s BlackBerry to a junior resident about going to the 7th floor. At 10:25, she makes another call on the senior’s BlackBerry. She hangs up at 10:25 and goes to see a patient on the 7th floor who looks like he is choking/having a seizure. At 10:26, senior asks a nurse for a Yonker for suction and attends to the patient. Junior resident arrives at the patient’s room. (Looks like an emergency situation: about 7 staff nurses and doctors were in the patient’s room.)

### Interruptions

Despite residents acknowledging that the use of smartphones had reduced the time spent on responding to informational messages, residents also perceived an increase in the overall number of messages and calls received. The substantial volume of interruptions was reflected in the number of phone calls and emails received as determined by both smartphone usage as well as by work shadowing residents (Table 2). From analyzing the frequency of calls and emails, a senior resident who usually carried both the team BlackBerry (point of contact and communication for nursing and allied health staff) and the senior BlackBerry (typically used for communication within the medical teams) would receive on average 11.6 calls and 25 emails within a 24-hour period. The volume of calls and emails could be higher during evenings and weekends when residents were covering for other teams and thus carrying multiple smartphones. (Textbox 2, data extract 1).

Interviews with the staff physicians also highlighted similar observations where physicians noticed the disruptions that the smartphone brought upon their teaching and patient care rounds (Textbox 2, data extract 2). From work-shadowing sessions, there were 4.6 average interruptions per hour for a resident when considering all direct calls, emails, and face-to-face communications (Table 2). These interruptions, however, were sometimes even more frequent. For example, within a 40-minute team meeting with the attending physician, 7 interruptions were observed. This included 5 direct phone calls to residents, where residents ended up taking the calls and leaving the room while the meeting was in progress (Textbox 2, data extract 3). In particular, numerous patient interactions were noted to be interrupted by direct calls. Often residents were observed answering phone calls on their smartphones, exiting the room, and then resuming patient interaction once the call was completed (Textbox 2, data extract 4). Residents did note that this had a negative impact on communication, especially during family meetings.

Considering these high volumes of communications, it is not surprising that residents commented that they felt overwhelmed by the constant interruptions, which may be a result of the increased availability of multiple communication options (Textbox 2, data extracts 5-7). Similar sentiments were echoed by the staff physicians, who observed that these interruptions could have detrimental impacts such as reduced downtime and impede residents’ abilities to provide patient care (Textbox 2, data extract 8).

**Interruptions: extracts from interviews and field notes**

Interview, resident 3 1. In July…and I carried six of [the BlackBerrys] and then there were no rules at all so nurses were just calling them and four would be going off at once and you couldn’t get anything done.

Interview, attending physician 3 2. So that intrusiveness I definitely find when we’re at the bedside, when we’re teaching, or even when we interact around cases and they go “‘Oh, I’m sorry. I better take this.”…So it’s a two-edged sword.

Field notes, work shadowing physician 2 3. At 3:04, attending for the team walks into the meeting room. At 3:07, junior resident’s BlackBerry rings. She picks up the smartphone and walks out to talk (sounds like a patient’s issue). She returns to the room at 3:10. At 3:13, team members go through patients’ cases with the social worker, pharmacist, and the attending physician. At 3:14, the team BlackBerry rings. Senior resident picks up. She looks at the BlackBerry and then starts calling back. As she walks out of the room, she says, “Hi, it is team (number). Who paged?” At 3:14, another junior resident’s BlackBerry goes off. He leaves the room but returns quickly.

Field notes, work shadowing physician 11 4. Senior resident returns to the patient’s room and continues examining her. While in the patient’s room, I (observer) could hear the resident talking on the BlackBerry. I asked her later what calls she had while in the room. It turns out she had 3 phone calls and 2 texts. Two of the calls were from the radiation oncologists and 1 call from the pathologist. She also received 1 text on the team BlackBerry and 1 text on the senior’s BlackBerry from the pharmacist.

Interview, resident 1 5. The only negative I can think of is just the incredible number of communications that you get, you know, text messages and emails and everything else. So just the number can sometimes be overwhelming.

Field notes, ward observations, March 18th 6. At 8:40, resident #1 talks to resident #2. Resident #1 complains that he got about 1000 pages after he had to take over.

Interview, resident 3 7. There was no choice [before]. Now there’s a choice to page; there’s a choice to text. You can ask for no response, email response, call back response, or call. So there’s six choices, right? There’s probably more but that’s sort of the ones that I’ve been using...

Interview, attending physician 3 8. I recognize that it comes at a significant cost…because other people are interrupting them and it probably comes at a personal cost to them in terms of the need for increased vigilance and attention and less downtime for them, like there are even more intrusions in their lives.

### Interprofessional Relationships

A strong theme that emerged throughout interviews was the impact of this new technology on interprofessional relationships. Nurses commented that the new system reduced opportunities for face-to-face interactions, which many valued. Nurses reported that they found it more difficult to build interprofessional relationships through the new technology. Specifically, the smartphones and use of email messaging reduced verbal conversations, which nurses felt prevented them from getting to know the residents, discouraged interest in their work, and reduced opportunities for nurses to have direct educational experiences with the residents (Textbox 3, data extracts 1 and 2). Additionally, nurses found the process depersonalizing to have the team BlackBerry as the primary point of contact instead of having direct interactions with the specific physician (Textbox 3, data extracts 2 and 3). 

In contrast, physicians perceived no major negative changes with this new technology. Since nurses were required to type in their names to send emails, physicians felt that they learned nurses’ names better and thus perceived their interprofessional relationships had actually improved (Textbox 3, data extracts 4 and 5).

**Interprofessional relationships: extracts from interviews and field notes**

Interview, nurse 9 1. With [general internal medicine] it’s really hard enough that the residents change every month or every 4 weeks so it’s hard to build that relationship with them in terms of what knowledge they have and even them knowing who we are. And then on top of that with the BlackBerry system…it is convenient, but in terms of building the team dynamics, because we are focused on interdisciplinary care, it’s hard to build that when a lot of it is through technology.

Interview, nurse 2 2. I know some of the nurses sort of have complaints or have concerns about the [new] paging [system], you lose the face-to-face communication, you lose getting to know the residents, really. Right now they just know everybody as a team. “‘Oh, I’ve sent the team this.” They miss that, especially the older nurses who are used to communicating face-to-face and getting to know the physicians more on a personal level.

Field Notes, ward observations, February 20th 3. Nurse types an email, “To team (number): Email response requested. Message: Thank you for replying back. Can I get your name so that I can write a verbal order in chart and how often to check [blood capillary glucose]? Patient said he doesn’t take insulin just the metformin and gliclazide. Thank you.”

Interview, resident 3 4. What’s great about the email system is that you have the nurse’s name cause it’s really hard because we work with a lot of nurses. And sometimes— I always introduce myself to the nurses, but they never give me their names back, so it’s nice to have that (BlackBerry) in front of you, and then if you forget the nurse you can just check the name again, so I find it much easier to work with people if you know their names.”

Interview, resident 4 5. You know, I’d actually say it’s maybe helped interaction with nurses only because when they [email] and they put their name. Like in the past, like it’s easy to not know nurses’ names because there’s so many of them, but when they’re emailing and they say like, you know, “Please reply to” and then it has the name like Joan, then you can go to the ward and say, “Hi Joan, I got your message. Thanks for sending it.” And I actually liked it. I got to know nurses’ names actually better through it…”

### Gap in Perceived Urgency

From our data analyses, we identified a gap in what physicians and nurses perceived as urgent patient issues. If a physician did not perceive the issue communicated to be urgent, often there would not be a response despite a request to respond having been made by the nurse, or the resident would reply with an email when the nurse requested a telephone call or otherwise.

#### Nurses

Nurses perceived both a lack of acknowledgment of messages as well as not receiving the requested response (Textbox 4, data extracts 1-3). This perception was confirmed by analyzing responses to nurses’ emails in which nurses actually only received an email response 50% of the time requested. They also felt many physicians ignore emails, similar to their ignoring pages previously. Nurses found it frustrating and felt belittled when physicians ignored their communications (Textbox 4, extract 4). With a lack of acknowledgment, nurses often felt the need to resend messages. Nurses also reported the need to have clear specifications of when and how to inform physicians such as for abnormal vital signs or laboratory results. It was observed that nurses would consult each other to see if physicians should be informed (Textbox 4, data extract 5).

#### Residents

Physicians also commented that there were too many direct calls for low importance items and a high number of emails that were of trivial importance (Textbox 4, data extract 6).

Much of this gap of perceived urgency may be attributed to the numerous methods that are now available to contact physicians. Though the smartphone system provides various options to contact residents, the array of choices often created confusion and a mismatch of responses among clinicians (Textbox 4, data extract 7).

**Gap in perceived urgency: extracts from interviews and field notes**

Interview, nurse 9 1. But, for example, if I need to get an order for a medication I would do an email, but if it’s something like the patient or the family wants to speak to the doctor and they really need a time right away or something urgent like that, I would ask them to call back. But then I find the doctors don’t always call back, anyways. They just use the email system.

Field notes, ward observations, Nov 25th 2. At 11:38, nurse 4 typing an email on computer #1: “Team (number), call back requested: I just received your order on (dosage) medication X. Would you like to discontinue the IV medication X? ”Page sent at 11:39am 30 seconds. At 12:10pm, nurse 4 approached me (observer) and told me her webpage for a callback was responded with an email from the doctor. She said she did not check her inbox. She was informed by another nurse who saw the email (because the message that nurse 4 sent had her name on it) and that’s how the other nurse knows to tell nurse 4.

Interview, nurse 3 3. Yeah, it makes me wonder that maybe I shouldn’t be sending that information or [laughs] if the patient wants something, for example, they want to see the doctor to discuss test results or something, and I don’t hear back, like even just a simple acknowledgment like, uh, “Okay, we’ll try to see them sometime this afternoon” or something would be nice instead of just sending something out into the void.

Interview, nurse 3 4. There’s nothing worse than, like, if I’m sending a message and I think it’s important…like it might not be that important to the physician ‘cause they know the case more in depth, but I might think it’s quite important, or it’s something that’s really important to the patient. But it’s sometimes hard, it’s hard to convey that urgency through a written message sometimes, and I don’t like to keep sending another page over and over again. It feels like I’m annoying the physician probably [laughs], but if I don’t get that response, it makes me second guess myself, like okay, I guess it doesn’t deserve a response or…

Field notes, ward observations, February 20th 5. Nurse E: “I got the hemoglobin results, it is X; should I email the team about it?” Nurse D says she should not and explains why.

Interview, resident 2 6. …I’ve been having some calls for bowel movement problems or for sleeping pills or something; very minor stuff that I think could at least be dealt with, with an email.

Interview, allied health 6 7. ...you know, there’s 500 ways to contact a person. You get their email, you get their phone number, you get their pager number, you get face-to-face. So I think to establish the best way to contact somebody, why not just face-to-face or BlackBerry, you know. You can use the phone on the BlackBerry, or you can email on the BlackBerry. I think there’s confusion in the unit on how to communicate or the best way to find certain people, and that’s when things take longer to happen…I think we’re given so many ways to communicate with people sometimes that we don’t know which one is the best one or the most efficient to get in touch with them.

### Professionalism

This theme focused on physicians’ behavior around the use of smartphones and how nurses, allied health professionals, and attending physicians perceived the manner in which residents handled interruptions by phone call or email message that could be regarded as unprofessional.

While having a conversation with a resident on the ward, nurses and other allied health professionals occasionally reported that the resident would answer a call on his or her smartphone, thus interrupting their conversation. Nursing and allied health professionals also found it disruptive when residents answered calls during interprofessional rounds (Textbox 5, data extracts 1 and 2).

Similarly, during patient rounds or educational rounds, attending physicians mentioned that they also found residents’ behavior with smartphones to be unprofessional at times (Textbox 5, data extracts 3 and 4). One attending physician observed that while smartphones increased the availability of the residents to other clinicians, it reduced the local availability of residents due to the constant distractions and interruptions from the device. In effect, the residents were made more global but less local (Textbox 5, data extracts 5 and 6). As noted by some, the continued calls and checking of messages often took away the quality time which residents spent interacting with the attending physician and their interprofessional colleagues.

Finally, this behavior was also observed during patients’ interactions, where residents would pick up phone calls, check, or type messages while talking with patients or supervising a procedure. Although patients’ perceptions were not obtained, other clinicians commented that such behavior could create negative perceptions among patients (Textbox 5, data extracts 7 and 8).

**Professionalism: extracts from interviews and field notes**

Interview, nurse 14 1. I did have a couple of doctors from other teams that were just constantly chatting on the phone while communicating with nursing staff. I found that to be kind of unprofessional.

Interview, allied health 6 2. In rounds or orientating new residents, I find that, yes, the BlackBerry does go off, and it rings, and it can interrupt face-to-face communication sometimes or that residents are checking emails as they’re talking to people face-to-face. So that’s also where you’re not sure if they’re really listening to what you’re saying or not.

Interview, attending physician 3 3. And sometimes it may indicate they’re being quite unsubtle about indicating that they’re not interested in being present all the time. That they’re bored. But I think it’s quite rude, and I think it’s undisciplined.

Interview, attending physician 1 4. It’s probably more annoying than anything. This is true of BlackBerrys, anyone using BlackBerrys. Anyone who has a BlackBerry will talk to you and look you in the eye and then kind of look down towards their BlackBerry.

Interview, attending physician 3 5. There’s definitely a convenience around, for example, their ability to be able to page other services, so if they need to get a hold of one of the consulting services they can page them and walk around and be able to pick it up instantly anywhere and not have to go back to the desk and things like that. But I think it also may limit the depth of their ability to interact with anybody else around them because they’re always sort of being distracted and it happens every single hour if not more. It happens constantly.

Interview, attending physician 3 6. And I think almost to some extent it’s an implicit permission that gets granted to the house staff to disrupt their own teaching experience and disrupt others around them because everybody is doing it, because everybody is being “BlackBerried.”

Interview, attending physician 3 7. I think most of the time, the BlackBerry is seen as a nuisance and a disruptive factor. And I think that most patients would not be terribly impressed no matter how much time you spent explaining why you need it.

Field notes, work shadowing physician 2 8. Senior walks out of the patient’s room while typing on the BlackBerry. She finishes typing and returns to the room at 5:36. Senior looks at her BlackBerry and starts typing inside the room in front of the patient. She pauses to look at the patient and the resident doing the procedure [paracentesis]. She resumes texting again and walks out of the room at 5:38. Another resident walks out, and senior speaks with the resident. Senior returns to the room and speaks with the patient. She asks the patient if he has ever gotten a successful tap before. Senior looks at her BlackBerry and starts typing.

## Discussion

### Principal Results

In this study, we described the nature of communications and the perceived impact of a new smartphone system for residents on busy general medical services. Smartphones were used frequently by residents to communicate about patients. We found that residents, nurses, and other clinicians perceived improvements in efficiency over the traditional paging system likely because smartphones appear to address many of the known issues with numeric paging such as inability to triage information or having to wait by a phone for a return call. The overall number of interruptions, however, was perceived to have increased likely because it was now easier for nurses to initiate communications. With more communications occurring over written modes such as emails and text messages, nurses perceived a negative impact to relationships with physicians because of decreased verbal conversations. Smartphone use in this study also highlighted the discordance between what nurses and physicians perceive as urgent. Finally, residents’ behavior when communicating with smartphones during patient care and education learning activities was perceived at times as unprofessional.

While the gap between what nurses and physicians perceive to be urgent has been previously identified [18], we found that the gap was likely made greater by increasing the number of methods for clinical communication. Email communication may also decrease verbal interactions—deemed by many clinicians as the most effective and optimal method of communication—which can result in the impediments of interprofessional collaboration and relationships [19]. Finally, the issue of digital professionalism is part of a larger issue of both knowing and educating medical trainees in the proper use of new media [20,21]. While there are established definitions of medical professionalism, these have not necessarily kept pace with the rapidly evolving new digital media [22-25]. Without being given formal guidance in using this new technology, residents appear to handle interruptions by trying to be efficient and minimize response times but at the expense of interprofessional and patient relationships.

**Figure 2:**
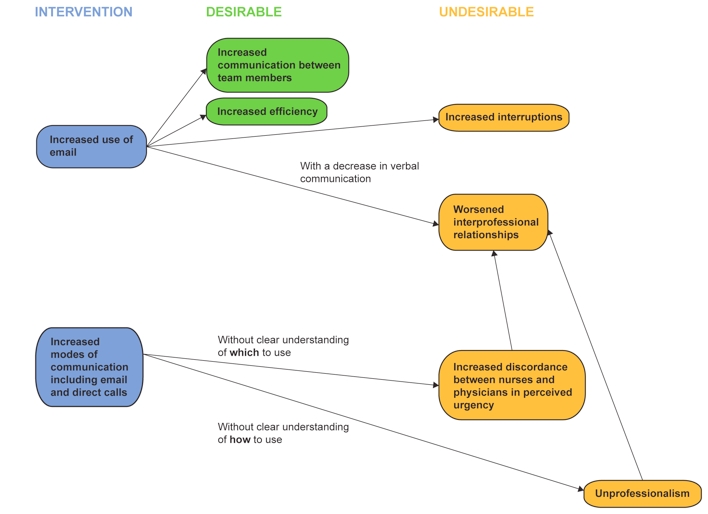
Potential positive and negative effects of changes to communication system in the use of smartphones

Our findings highlight the important aspects to consider when implementing systems to improve clinical communication. While increasing overall team communication and efficiency are important goals, it is also equally critical to consider the range of possible and potential impacts as unintended consequences can occur (Figure 2). Considering these themes (efficiency, interruptions, interprofessional relationships, common understanding of urgency, and professionalism) as a framework could aid in the design and evaluation of communication systems. This may facilitate the development of communication systems in which the appropriate information is sent through the appropriate medium with the appropriate intrusiveness, while incorporating processes that foster interprofessional relationships and promote digital professionalism.

As shown in Figure 2, our findings suggest that email or text conversations appear to create the desirable and undesirable effects as listed above. The increased modes of communication when moving from pagers to smartphones include both text messages as well as direct phone calls. Our findings support that increased modes of communication appear to highlight the gap in perceived urgency and highlights professionalism issues.

### Comparison With Other Work

The issues with numeric paging have been well documented. These include a high number of interruptions with an average of 9 pages per hour on some clinical services [26]. While all pages interrupt, only 30% have been found to require urgent attention, and the majority do not require a response within an hour [27]. As well, one study found that 14% of pages are sent to the wrong physician, with 47% of those pages requiring urgent attention [7]. The response rate for pages in one observational study was 90% [4]. 

To our knowledge, this is the first comprehensive mixed-methods evaluation of the use of smartphones on hospital communications. Studies using surveys have found perceived improvements with the use of smartphones among clinicians [15,16]. Through the use of ethnography observations and interviews, we were able to determine *how* smartphones are used and their impacts on different domains including unintended effects.

### Limitations

There were limitations to this study. Smartphones were provided only to residents, and the behavior and perceptions would likely be different if other professions were provided with smartphones. The study was also conducted at two hospital sites; thus, generalizing to other institutions with different hospital cultures may yield different results. However, our intervention used standard components of smartphones and email with minimal customization, and other academic hospitals may be able to learn from this experience. Future studies could look at other measurements, outcomes, and impacts on quality of patient care such as time to resolution of urgent items or the effect of interruptions on patient care.

### Conclusions

Routine adoption of smartphones by residents appeared to improve efficiency over the use of pagers for physicians, nurses, and allied health professionals. This was balanced by negative communication issues of increased interruptions, a gap in perceived urgency, weakened interprofessional relationships, and unprofessional behavior. Further communication interventions are required that balance efficiency and interruptions while maintaining or even improving interprofessional relationships and professionalism.
